# The Use of TSH in Determining Thyroid Disease: How Does It Impact the Practice of Medicine in Pregnancy?

**DOI:** 10.1155/2013/148157

**Published:** 2013-05-09

**Authors:** Offie P. Soldin, Sarah H. Chung, Christine Colie

**Affiliations:** ^1^Georgetown University School of Medicine, Georgetown University Medical Center, Washington, DC 20057, USA; ^2^Departments of Oncology, Medicine, Pharmacology, and Physiology, Georgetown University Medical Center, Washington, DC 20057, USA; ^3^Departments of Obstetrics and Gynecology, Georgetown University Medical Center, Washington, DC 20057, USA; ^4^Department of Oncology, Lombardi Comprehensive Cancer Center, Georgetown University Medical Center, LL, S-166, 3800 Reservoir Road NW, Washington, DC 20057, USA

## Abstract

During the last four decades, there have been considerable advances in the efficacy and precision of serum thyroid function testing. The development of the third generation assays for the measurement of serum thyroid stimulating hormone (TSH, thyrotropin) and the log-linear relationship with free thyroxine (T4) established TSH as the hallmark of thyroid function testing. While it is widely accepted that TSH outside of the normal range is consistent with thyroid dysfunction, a vast multitude of additional factors must be considered before an accurate clinical diagnosis can be made. This is especially important during pregnancy, when the thyroid is under considerable additional pregnancy-related demands requiring significant maternal physiological changes. This paper examines serum TSH measurement in pregnancy and some associated potential confounding factors.

## 1. Introduction

TSH is a 28-kDa glycoprotein released from thyrotrophs in the anteromedial region of the pituitary gland that stimulates thyroidal thyroxin (T4) and triiodothyronine (T3) synthesis [1]. There is a strong inverse log-linear relationship between serum TSH and serum-free T4 concentrations. Small changes in T4 concentrations will provoke very large changes in serum TSH. The diagnostic superiority of TSH measurement arises principally from this inverse log/linear relationship between circulating TSH and free T4 concentrations [2–4]. Serum TSH concentrations are considered the most reliable indicator of thyroid function abnormalities, and TSH analysis stands as the primary means of studying thyroid function [2, 5, 6]. However, clinicians should be aware of certain conditions when TSH analysis results may be incorrect due to assay inaccuracies. Because it is trusted among clinicians in identifying thyroid disease, it is important to recognize several physiological states in which serum TSH concentrations may not be consistent with the clinical presentation, and may provide misleading results. Specifically, pregnant women exhibit a different thyroid function profile, particularly during the first trimester, necessitating pregnancy-specific reference intervals. In addition, since thyroid and pituitary functions are not stable in pregnant women, measuring TSH may not be sufficient for the assessment of thyroid function during gestation.

We discuss relevant information on analytical, as well as clinical, aspects of serum TSH determination and its usefulness in detecting subtle thyroid function abnormalities associated with the pregnant state. Since a single test can reliably indicate thyroidal status, it would be beneficial if all pregnant women would undergo serum TSH measurement as soon as pregnancy is established.

## 2. Laboratory Serum TSH Analysis

The current standard of care calls for the use of the third generation TSH assays with functional sensitivity of <0.02 mIU/L [2, 7–9], a level of sensitivity necessary for detecting degrees of TSH suppression. The current third generation chemiluminescent immunoassays provide excellent sensitivity and specificity as well as the necessary low limits of detection and quantification.

Serum TSH testing provides better sensitivity for detecting thyroid dysfunction than do the current indirect free T4 tests using immunoassays [2, 9–11]. Although methods to directly measure free T4 and free T3 using liquid chromatography tandem mass spectrometry (LC/MS/MS) are currently available in reference laboratories, they are not readily available for most practicing clinicians. This is a costly technology; requiring the separation of T3/T4 from their binding proteins by using equilibrium dialysis or ultrafiltration prior to the spectrometry. A highly trained and dedicated operator is required, and the process can be time consuming.. Importantly, the use of LC/MS/MS for serum thyroid hormone measurements provides results that have higher sensitivity and specificity and are undoubtedly the wave of the future. However, TSH is a large molecule, too large for current methods of analysis using LC/MS/MS. Efforts are being made to find a shorter more specific fragment of TSH measurable by LC/MS/MS. 

## 3. Confounding Factors in the Measurement of TSH

TSH laboratory assays vary in their susceptibility to assay interference [12], and there are several clinical situations in which the measurements of serum TSH alone may yield misleading results (Table 1). A physician may suspect assay interference when a reported value is inconsistent with the clinical status of a patient. Clearly, without such a physician's inquiry, it is difficult for the laboratory to proactively detect assay interference from a single measurement such as an isolated TSH test. The most practical way to investigate a suspected interference is to test the specimen by a different manufacturer's method and check for discordance between the test results. Occasionally, a biological check can be made using TRH-stimulation or thyroid hormone suppression to validate a suspected inappropriate serum TSH level. Interferences producing a falsely elevated TSH value will usually be associated with a blunted (<2-fold increase) response to stimulation. Some causes of elevated TSH are listed in Table 2.

Some examples of potential TSH assay interference include (1) assay cross-reactivity, (2) heterophile (animal) antibody interference with TSH assay reagents, including human anti-mouse antibodies (HAMA), (3) endogenous antibodies to TSH, and (4) *in vivo* or *in vitro* drug interactions.

*(1) Cross-Reactivity.* In general, the specificity of an immunoassay depends on the ability of the antibody reagent to discriminate flawlessly between the analyte and structurally related ligands. The use of monoclonal antibodies for developing TSH immunoassay methods has virtually eliminated previously experienced cross-reactivity problems with other glycoprotein hormones such as LH or hCG that plagued the early TSH radioimmunoassay methods. However, because each monoclonal antibody differs in its specificity for recognizing various circulating TSH isoforms, these differences in assay-specific antibodies can result in the reporting of TSH values that may differ by as much as 1.0 mIU/L for a given serum sample [13].

*(2) Heterophile Antibodies/Human Anti-Mouse Antibodies (HAMA).* Heterophile antibodies represent a group of relatively weak multispecific, polyreactive antibodies with specificity for poorly defined antigens that react with immunoassays derived from two or more species [14, 15]. Most frequently, such heterophile antibody interferences result from IgM rheumatoid factor or HAMA. Immunometric assay methods that use monoclonal antibodies of murine origin are more prone to HAMA interference than competitive immunoassays and create a signal that results in a reported falsely high value [16]. Such HAMA interferences can produce inappropriately normal values in patients with clinical disease [17]. Despite the measures used by manufacturers to neutralize interferences, both the clinician and the laboratory must be aware of this possibility when an apparently inappropriate test result is encountered.

*(3) Endogenous Antibodies.* Similarly, endogenous antibody interferences are characterized by either falsely low or falsely high TSH values, depending on the type and composition of the antibody assay employed.

*(4) Drug Interferences.* Certain medications may interfere either *in vitro* or *in vivo* with the measurements of serum TSH concentrations [18, 19]. Drugs can have *in vitro* effects if serum samples contain sufficient concentrations of certain therapeutic and diagnostic agents to produce methodological interference. A number of drugs cause hypothyroxinemia in euthyroid patients by decreasing thyroxine binding globulin (TBG) concentrations (androgens; niacin), decreasing T4 binding to TBG (high dose salicylates, phenytoin, and carbamazepine), and/or increasing T4 metabolism (carbamazepine, phenobarbital, and phenytoin). Other drugs can lead to hyperthyroxinemia in euthyroid patients by increasing TBG concentrations resulting in higher total T4 concentrations and lower free T4 (clofibrate, estrogen, 5FU, and heroin/methadone) [18–20]. Drugs such as amiodarone, iopanoic acid, high-dose propranolol, and nadolol may raise circulating T4 levels by inhibiting the conversion of T4 to T3. Glucocorticoids can have *in vivo* effects on thyroid function by altering TSH, thyroid hormone secretion, and/or thyroid hormone metabolism [18, 19]. Therefore, any concomitant use of medication should always be carefully taken into account when a TSH laboratory result may not fit with a clinical presentation and before taking interventional steps. 

## 4. Biologically Active TSH versus Immunoactivity of TSH 

High concentrations of serum TSH may be the result of the rare presence of biologically inactive forms of TSH resulting from pituitary-hypothalamic disease. In such individuals, the basal TSH levels, if measured by an immunoassay, will have elevated TSH concentrations, yet when measured by a cytochemical bioassay will be found to be normal [21]. This finding, coupled with the absence of the normal rise of thyroid hormones in response to thyrotropin-releasing hormone- (TRH-) mediated release of TSH, will confirm the secretion of Bioinactive TSH. Primary thyroid disease as a cause for the elevated immunoreactive TSH can be excluded by the absence of circulating thyroid antibodies and by a normal thyroidal radioiodine uptake response to exogenous TSH. In patients with idiopathic central hypothyroidism due to biologically inactive TSH, there is an excess of circulating TSH-beta. In this case, TRH implies the secretion of TSH of full biological potency [22, 23].

## 5. Changes in Thyroid Physiology during Pregnancy

During pregnancy, thyroid function tests most often reflect normal physiological changes that occur during a period of high metabolic demand. The placenta secretes high levels of human chorionic gonadotropin (hCG), a glycoprotein with a common alpha-subunit and considerable homology with the beta-subunit of TSH. Thyroid function is increased due to activation of the TSH receptor by hCG. Immunometric TSH assays have overcome the problems posed by hCG cross-reactivity [24]. However, hCG has weak thyroid-stimulating activity, and levels of serum hCG will increase during the postfertilization period, peaking at 10–12 weeks of gestation [25]. During this time, serum T3 and T4 concentrations can be elevated, while TSH concentrations will be reduced [26]. In up to 10–20% of normal pregnant women, serum TSH concentrations are transiently low or undetectable [27]. In a report of 63 women with hCG concentrations of greater than 200,000 IU/L, 67% of the samples displayed markedly decreased serum TSH levels of less than 0.2 mIU/L, and 32% of the samples showed serum-free T4 greater than 1.8 ng/dL. All women exhibiting serum hCG greater than 400,000 IU/L demonstrated suppressed serum TSH concentrations [28]. These findings were transient and lasted for the first three months of pregnancy. However, TSH concentrations lower than the low reference range for healthy nonpregnant women should be considered to be a normal finding during the first trimester. 

Very early following conception, thyroid function is further enhanced due to the rise in estrogen, leading, as early as the first six weeks of pregnancy, to an increase in serum TBG concentrations. Additionally, pregnancy-related TBG sialylation results in a decrease in the clearance of TBG [29]. To meet the new needs, the thyroid gland increases production of T4 and T3, and laboratory findings often indicate a 50% increase in TBG concentrations and a similar increase in total T4 and T3 concentrations during the first trimester that plateau at approximately 20 weeks of gestation [29]. A new steady state is reached by the second trimester, and the production of thyroid hormone returns to pre-pregnancy rates.

## 6. TSH Assessment in Pregnant Women

Normal thyroid function is imperative for optimal maternal health and fetal neurodevelopment. Since the occurrence of disorders of the thyroid gland are relatively frequent in women of childbearing age, TSH measurements are useful in detecting subtle thyroid dysfunction associated with poor pregnancy outcome [26]. The enhanced sensitivity of the third generation assays has established the lower TSH limit for nonpregnant women as approximately 0.3 mIU/L and affords the accurate detection of the low serum TSH values often seen during the first trimester. The new clinical practice guidelines for the management of thyroid dysfunction during pregnancy and postpartum published by the American Thyroid Association [30] and the recent Endocrine Society clinical practice guidelines [31] recommend the use of trimester-specific reference intervals for TSH. Further, the recommendation is to use trimester-specific reference intervals defined in populations with optimal iodine intake. It is further recommended by the ATA that if trimester-specific reference intervals for TSH are not available in the laboratory, then TSH intervals should be 0.1–2.5 mIU/L for the 1st trimester, 0.2–3.0 mIU/L for the 2nd trimester, and 0.3–3.0 mIU/L for the 3rd trimester (Table 3).

In the case of women with autoimmune thyroid disease, it is important to note that thyroid autoimmune activity decreases during pregnancy. The recommendation is to assess trimester-specific free T4 combined with TSH. Measurement of antithyroid peroxidase antibodies (TPOAb) and/or TSH receptor antibodies (TSHRAb) adds to the differential diagnosis of autoimmune thyroid disease (AITD) and nonautoimmune thyroid diseases [32].

Access to a broad spectrum of thyroid function tests must be considered a prerequisite for taking proper care of pregnant women with AITD. Due to the high TBG concentrations, the best laboratory assessment of thyroid function also in AITD is a free thyroid hormone estimate—in hypothyroidism a serum-free T4 estimate combined with TSH and in hyperthyroidism-free T4 and T3 concentrations combined with TSH. These free thyroid hormone measurements do not always correct completely for the binding protein abnormalities. Thus, if in doubt, samples should be measured in another laboratory with different platforms for free thyroid hormone measurements or combined with total hormone measurement. Measurement of TPOAb, TgAb, and/or TSHRAb will add to the differential diagnosis between AITD and nonautoimmune thyroid disease. Thus, presence of TPO antibodies very often predicts the risk of hypothyroidism, and, in pregnant women with low serum TSH concentrations, hyperthyroidism will be predicted by TSHRAb in 60–70% of the cases.

Moreover, in the case of maternal hypothyroxinemia (low free T4 with normal TSH), which is increasingly recognized for its association with neurodevelopmental deficits, TSH is within the normal range and is therefore not useful in detecting this problem [33].

Measurement of serum-free T4 concentrations in the dialysate or ultrafiltrate of serum samples using liquid chromatography/tandem mass spectrometry has proven to be the most reliable measurement of free T4 during pregnancy, which typically decreases with advancing gestational age, specifically during the transition from first to second trimester [34, 35]. This is also the optimal method to assess serum free T4 during pregnancy is measurement of T4 recommended by the 2011 ATA clinical guidelines for the treatment of thyroid disease in pregnancy [30].

Tandem mass spectrometry, however, is relatively expensive, and, unfortunately, it is not readily available in most clinical situations. Thus, an alternative is to utilize the trimester-specific immunoassays realizing that the results are often higher due to pregnancy-related issues. Free T4 assays oftentimes fail to be as reliable owing to variable increases in TBG and decreases in albumin levels which take place during pregnancy [36]. Therefore, if free T4 measurement by LC/MS/MS is not available, clinicians should use whichever measure or estimate of free T4 is available in their laboratory, being aware of the limitations of each method. Serum TSH is a more accurate indication of thyroid status in pregnancy than any of these alternative methods. There are others who claim that if abnormalities are suspected, alternatively, the analysis of serum total T4 levels, which are more reliable during pregnancy, can be used to assess thyroid function [31].

## 7. Additional Considerations in TSH Assessment

### 7.1. Diurnal Variation

Serum TSH normally exhibits a diurnal variation with lowest serum concentrations detected between 1:00 and 16:00 hours and a peak in serum TSH concentrations between 00:00 and 04:00 hours [37]. This variation should not influence the diagnostic interpretation of test results since most clinical TSH measurements are performed on ambulatory patients between 08:00 and 18:00 hours. In like manner, the reference intervals for TSH are typically established using specimens collected during similar times during the day. Furthermore, with a half-life of approximately 7 days, serum T4 concentrations do not change sufficiently in one day to raise TSH secretion; there is no need to withhold LT4 therapy on the day of blood testing for TSH [2]. 

### 7.2. Hospitalized Patients

Nonthyroidal illnesses can frequently alter thyroid hormone peripheral metabolism and hypothalamic-pituitary-thyroidal (HPT) function. This results in thyroid test abnormalities, including both increased and decreased serum TSH levels [38]. It is important to distinguish the generally mild, transient TSH alterations typical of NTI from the more profound and persistent TSH changes associated with hyper- or hypothyroidism [39]. 

### 7.3. Intraindividual Variability

One would be remiss to exclude the mention of the fact that population-based reference intervals include not only between-individual variation, but also within-individual variation as well [3, 40]. In the case of TSH it is very important to note that within-person TSH variability is relatively narrow and varies by only 0.5 mIU/L when tested monthly over a one-year span. This is a narrow interval considering that the *between-person variability *is more variable resulting in the 95% confidence interval of 0.3 to 3.0 mIU/L [41–43]. Consequently, it is highly possible that abnormal test results for a single individual may go largely undetected if the results remain within the normal range for the wider population—in fact, when the index of individuality for a thyroid test is below 0.6, population-based reference intervals are fairly unreliable at gauging individual change [44]. This places limits on the usefulness of population-based reference intervals for detecting thyroid dysfunction in individuals [3, 40]. Theoretically, it may be important to evaluate individuals with marginally, although confirmed, low (e.g., 0.3-0.4 mIU/L) or high (3.0–4.5 mIU/L) TSH levels relative to patient-specific risk factors for cardiovascular disease, rather than relative to the normal TSH reference interval [45]. However, there are no data to show increased morbidity and mortality in individuals with serum TSH levels that are not within a person's individual range but still fall within the population reference interval.

## 8. TSH Trimester-Specific Reference Intervals 

Thyroid disorders are relatively frequent in women of childbearing age. Moreover, overt and subclinical hypothyroidism and hyperthyroidism are associated with poor pregnancy outcome. Therefore, the correction of maternal thyroid dysfunction during all stages of pregnancy is very important for the health outcome for both mother and fetus.

 As discussed, serum TSH provides the most sensitive test to reliably detect thyroid function abnormalities. During pregnancy the lower and upper reference limits for serum TSH are decreased by about 0.1-0.2 mIU/L and 1.0 mIU/L, respectively, compared to the TSH reference interval of 0.4–4.0 mIU/L of nonpregnant women [46]. The Endocrine Society and the most recent American Thyroid Association (ATA) guidelines recommend using a TSH upper limit value of 2.5 mIU/L for preconception and the first trimester and 3.0 mIU/L for the second and third trimesters [11]. In accordance with the new ATA guidelines for women in pregnancy, trimester-specific reference intervals for TSH, as defined in populations with optimal iodine intake, should be applied, even though many commercial laboratories still do not provide these reference ranges [47]. 

Population studies have demonstrated the lower limit for TSH in first-trimester healthy pregnant women ranging from 0.03 to 0.10 mIU/L [46, 48–51]. It is important to mention that in the process of defining population-based trimester-specific reference intervals only women who are iodine sufficient and who do not have TPO antibodies should be included in the standardizing population. Due to the low intraindividual variation of serum thyroid hormones it would be ideal to use the individual's own reference interval of thyroid hormones, when diagnosing hypo- or hyperfunction, but this is rarely available.

## 9. Hypothyroidism in Pregnancy

 Hypothyroidism in pregnancy is linked to an increased rate of spontaneous abortion in pregnancies less than 20 weeks of gestation, IUGR, and in small for gestational age births. This, coupled with hypothyroid women oftentimes being anovulatory, contributes to the rarity (0.3–0.5% of screened women) of overt hypothyroidism being seen during pregnancy [52]. Subclinical hypothyroidism, defined by an elevated TSH level but normal free T4, is more commonly seen (2.0–2.5% of screened women) [53]. Thyroid peroxidase antibodies are found in 5–15% of women of childbearing age and account for the main cause of hypothyroidism seen in pregnancy [31]. Iodine deficiency is also a major cause for hypothyroidism in pregnancy.

The diagnosis of overt hypothyroidism in pregnancy is defined by decreased serum-free T4 concentration (using pregnancy-specific reference intervals) coupled with increased trimester-specific serum TSH concentrations. Early maternal low free T4 concentrations have been associated with a lower developmental index in children at 10 months of age, and children born to mothers with persistently low free T4 levels past 24 weeks showed marked deficits in motor and mental development. However, following levothyroxine supplementation, if free T4 levels were remedied during gestation, infants proceeded to have normal development, suggesting that specific timing and prolonged duration of low maternal free T4 were required for impaired neural development [54]. Therefore, it is recommended that maternal serum TSH concentrations should be measured approximately every 4 weeks during the first trimester and TSH should be checked at least once in the second half of gestation and at 6 weeks postpartum.

Despite this, maternal TSH levels, even in the upper range of normal for a given trimester, have been associated with increased rates of fetal loss [55]. Thus, it is essential to identify hypothyroidism promptly so that levothyroxine treatment can be provided throughout the remainder of the pregnancy. Women at risk for iodine deficiency or with family history of thyroid disease or recurrent miscarriage and infertility should be screened for hypothyroidism at the start of prenatal care [31].

Clinicians should be aware of the potential increased risk of adverse outcomes associated with subclinical hypothyroidism. A randomized control trial conducted in Italy demonstrated that levothyroxine intervention resulted in a reduction in adverse pregnancy outcomes in women with subclinical hypothyroidism and TPO antibodies. There have been no subsequent prospective randomized studies confirming or refuting this finding. However, this is the only RCT study to date, and therefore there is insufficient evidence to recommend for or against treatment. It is reasonable, however, to consider levothyroxine for the treatment of maternal subclinical hypothyroidism under these circumstances. The ATA clinical guidelines for women in pregnancy recommend that women with subclinical hypothyroidism in pregnancy who are not initially treated should be monitored for progression to overt hypothyroidism with a serum TSH approximately every 4 weeks until 16–20 weeks of gestation although this approach has not been prospectively studied [11].

Due to the thyroid-pituitary instability during pregnancy and to serum TSH suppression during the high peak of hCG at the end of the first trimester, TSH is insufficient as a sole and first-line diagnostic variable in determining maternal thyroid disease during the first trimester. Both total and free serum thyroid hormones are liable to false results during pregnancy. Therefore, when there is any suspicion of hypo- and hyperthyroidism that was not reflected in the laboratory, test results should be supplemented with TPOAb analysis in case there is suspected hypothyroidism and TSHRAb when hypo- or hyperthyroidism is suspected. 

## 10. Hyperthyroidism in Pregnancy

The most common cause of hyperthyroidism in pregnancy is Graves' disease. 95% of these patients will have thyrotropin receptor antibodies (TSHRAb) and thus thyrotropin-binding inhibitory immunoglobulin assays can be used to diagnose Graves' disease during pregnancy, as radioiodine administration is contraindicated. Overt hyperthyroidism is defined by a suppressed or undetectable serum TSH concentrations lower than 0.1 mIU/L and elevated serum T4 and T3 concentrations. If serum TSH concentrations are below <0.1 mIU/L, serum-free T4 and T3 levels should be measured. If the latter values are incongruent with serum TSH concentrations and/or clinical findings, total T4 should be obtained [11]. Normal serum free T4 concentrations despite low serum TSH levels define subclinical hyperthyroidism, which is not associated with adverse gestational outcomes [47]. Healthy pregnant women may exhibit serum TSH concentrations as low as 0.03–0.1 mIU/L, while overt hyperthyroid pregnant women will exhibit exceedingly low serum TSH concentrations (<0.01 mIU/L) [46, 48–51]. Overt hyperthyroidism is rare in pregnancy, occurring in only 0.1–0.4 percent of pregnant women [31]. Hyperthyroidism during pregnancy is associated with adverse outcomes such as spontaneous abortion, premature labor, low birth weight, stillbirth, preeclampsia, and heart failure [56, 57]. Rarely, labor, infection, preeclampsia, or cesarean delivery can precipitate thyroid storm. 

## 11. Conclusions

The measurement of serum TSH concentrations is considered the most reliable and sensitive indicator of thyroid function in nonpregnant individuals and during pregnancy due to its inverse logarithmic relationship with serum-free T4 concentrations. TSH assay interference may arise in instances of cross-reactivity with glycoproteins, endogenous TSH antibodies, heterophile antibody interference with assay reagents, or drug interactions. Factors such as laboratory assessment, age, sex, ethnicity, diet, education level, medications, socioeconomic status, body mass index, and smoking may affect thyroid function, resulting in an elevated TSH. It is, therefore, essential to be mindful of the interplay of variables on thyroid function and proceed accordingly in terms of treatment and diagnosis. It is essential to identify discrepancies in TSH measurement to ensure accuracy in the diagnosis of thyroid disease, particularly in pregnancy when adequate levels of thyroid hormone are vital to fetal neurodevelopment.

During normal pregnancy, the thyroid gland increases its activity to maintain appropriate concentrations of free T4 and T3. Activation of the TSH receptor by pregnancy-related elevations in hCG during the first trimester can also result in activation and increased thyroidal activity and a decrease in TSH reference intervals during the first trimester. These pregnancy-related changes in maternal normal reference intervals for TSH, free T4, and free T3 obviate the need for trimester-specific reference intervals for serum TSH and should be used in identifying overt hyper- or hypothyroidism in pregnancy. Other laboratory tests, such as detection of thyroid peroxidase antibodies or free T4 measurement using liquid chromatography/tandem mass spectrometry, can also provide useful information in identifying true thyroid abnormalities during pregnancy.

## Figures and Tables

**Table 1:** Clinical situations in which measurements of serum TSH alone may yield misleading results.

Condition	Serum TSH	Consequences of Clinical Action based on Serum TSH value Alone	Serum FT4
Heterophile antibodies	Normal	Failure to diagnose thyrotoxicosis	High
Central hypothyroidism	Normal*	Failure to diagnose hypothyroidism and investigate hypothalamic-pituitary structure function	Low
TSH-secreting pituitary adenoma	Normal*	Failure to diagnose thyrotoxicosis and investigate pituitary structure and function	High
Thyroid hormone resistance	Normal*	Failure to recognize the condition	High
Poor compliance with T4 therapy	High	Inappropriate increase in dose of T4	High
Delayed recovery of TSH secretion after treatment of hyperthyroidism	Normal or low	Failure to diagnose impending hypothyroidism	Low

*Serum TSH concentrations may also be high in these conditions, which should prompt measurements of serum FT4 and further investigation.

**Table 2:** Causes of elevated serum TSH concentrations.

Assay-related Bioinactive TSH secretion Heterophile antibodies	
Dysfunctions of the thyroid gland Family history of thyroid disease (latent thyroid disorder) TSH resistance syndromes Thyroid hormone resistance Germline mutations of TSH receptor Hashimoto thyroiditis Other autoimmune conditions Recovery phase of subacute thyroiditis	
Dysfunctions of the pituitary gland Pituitary tumors (TSH producing)	
Environmental Pregnancy Iodine deficiency Radioactive iodine treatment Medications (steroids, dopamine, iodine, amiodarone) Nonthyroidal illness Insufficient medication in individuals with a thyroid disorder	

**Table 3:** Trimester-specific reference intervals for TSH (mIU/L) [30].

First trimester	0.1–2.5
Second trimester	0.2–3.0
Third trimester	0.3–3.0
